# Mental healthcare for adults with mild intellectual disabilities: population-based database study in Dutch mental health services

**DOI:** 10.1192/bjo.2023.31

**Published:** 2023-03-03

**Authors:** Katrien P. M. Pouls, Maarten Cuypers, Mathilde Mastebroek, Jannelien Wieland, Monique C. J. Koks-Leensen, Geraline L. Leusink, Willem J. J. Assendelft

**Affiliations:** Department of Primary and Community Care, Radboud University Medical Center, The Netherlands; Curium LUMC, Leiden University Medical Center, The Netherlands; and Poli+, Leiden, The Netherlands

**Keywords:** Intellectual disability, mental health services, medical record, care provided, mental health disorder

## Abstract

**Background:**

Adults with mild intellectual disability (MID) experience more mental health disorders than the general population. However, mental healthcare may be insufficiently tailored to match their needs. Detailed information is lacking regarding care provided to people with MID in mental health services.

**Aims:**

To compare mental health disorders and care provided to patients with and without MID in Dutch mental health services, including patients with missing MID status in the service files.

**Method:**

In this population-based database study, we used a Statistics Netherlands mental health service database, containing health insurance claims of patients who utilised advanced mental health services in 2015–2017. Patients with MID were identified by linking this database with Statistic Netherlands’ social services and long-term care databases.

**Results:**

We identified 7596 patients with MID, of whom 60.6% had no intellectual disability registration in the service files. Compared with patients without intellectual disability (*n* = 329 864), they had different profiles of mental health disorders. They received fewer diagnostic (odds ratio 0.71, 95% CI 0.67–0.75) and treatment activities (odds ratio 0.56, 95% CI 0.53–0.59), and required more interprofessional consultations outside of the service (odds ratio 2.06, 95% CI 1.97–2.16), crisis interventions (odds ratio 2.00, 95% CI 1.90–2.10) and mental health-related hospital admissions (odds ratio 1.72, 95% CI 1.63–1.82).

**Conclusions:**

Patients with MID in mental health services have different profiles of mental health disorders and care than patients without intellectual disability. In particular, fewer diagnostics and treatments are provided, especially in those with MID with no intellectual disability registration, putting patients with MID at risk of undertreatment and poorer mental health outcomes.

Up to a third of adults with mild intellectual disability (MID), characterised by a significant deficit in intellectual (IQ range 50–70) and adaptive functioning,^1^ experience mental health disorders. This is double the general population estimates.^2^ The combination of MID and mental health disorders results in poorer general health outcomes, such as more all-cause hospital admissions and emergency department visits, compared with MID or mental health disorders alone.^3,4^ A lack of high-quality research on appropriate (mental health) care for this specific patient group contributes to this health disparity.^5^

There are several reasons for concern regarding the quality of care for patients with a combination of MID and mental health disorders in mental health services. First, either the MID or the mental health disorder may often be missed when symptoms are attributed exclusively to either of these specific disorders despite both states being present, so-called diagnostic overshadowing.^6,7^ Second, mental health service professionals perceive a lack of knowledge and experience in treating patients with a combination of MID and mental health disorders.^8,9^ Third, there is little research on how mental healthcare should be organised and provided to people with MID.^5^ In mental health services, patients with intellectual disability may be excluded from certain treatments or even any care at all, because the organisation lacks knowledge and expertise.^4,8^ Consequently, patients with intellectual disability experience long waiting times because of the scarcity of mental health services specialised in patients with intellectual disability.^4^ Fourth, patients with MID report negative experiences with mental health services, including poor accessibility and information provision.^5,10^ Finally, detailed information is lacking regarding the characteristics of mental health disorders and care provided to people with MID in mental health services. This also applies to those patients whose MID is potentially missed, and whose specific needs are thus a blind spot for mental health service professionals. Such information can give guidance on improving mental healthcare for people with MID and future research. The aim of this paper is to provide an overview of the prevalence of the range of mental health disorders and the mental healthcare provided to people with and without MID in mental health services, including those patients whose MID is not recorded in mental health service files.

## Method

### Study design and data source

This population-based database study investigated the prevalence of a range of mental health disorders and care provided to all patients utilising advanced mental health services in The Netherlands between 1 January 2015 and 31 December 2017. Information was retrieved from health insurance claims, which are collected in a central database at Statistics Netherlands, the Dutch national statistics office.^11^ In The Netherlands, mental health services are subdivided into basic mental health services for mild and low-complex mental health problems, and advanced mental health services for more severe and complex mental health disorders.^12^ Both types of mental health services are accessible to all patients after assignment and referral by a general practitioner, and all costs involved are covered by mandatory health insurance. The two mental health services submit their health insurance claims in different ways, with the claims from advanced mental health services containing much more detailed information about diagnosis and treatment versus the claims from basic mental health services. Health insurance claims submitted by advanced mental healthcare providers were collected and processed in a standardised manner, and were completed and available at Statistics Netherlands for research purposes in the Statistics Netherlands mental health (SN-MH) service database for the years 2015–2017. This study focused on patients with more severe and complex mental health disorders in advanced mental health services, which are referred to as mental health services in the rest of this article.

**Fig. 1:**
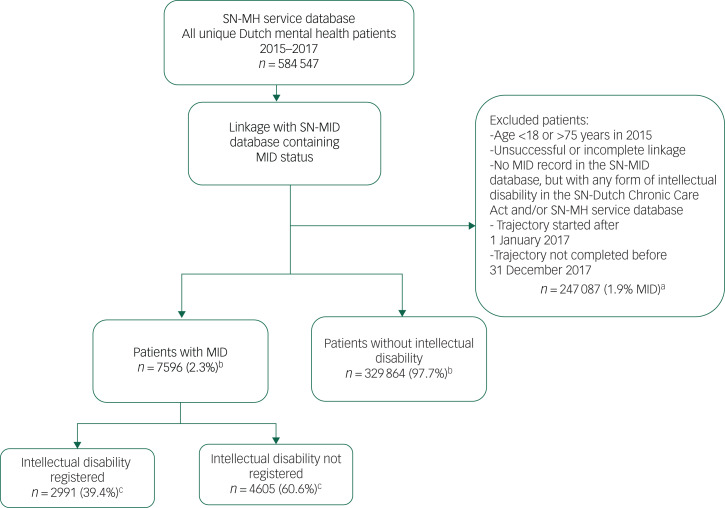
Flow chart of study sample selection. ^a^ Percentage of excluded patients with an MID record in the SN-MID database. ^b^ Percentage of included patients with an MID. ^c^ Percentage of patients with MID with or without an intellectual disability registration in the SN-MH service database. MID, mild intellectual disability; SN-MH, Statistical Netherlands Mental Health; SN-MID, Statistical Netherlands Mild Intellectual Disability.

In the mental health service files, an intellectual disability can be reported as a contributing diagnosis. To take potential under-recognition and under-reporting of MID in mental health services into account,^7^ the mental health service database was linked to a combined social services and long-term care database that included all users (in 2015) of services under the Chronic Care Act, the Disability Benefit Act or the Sheltered Employment Act, and for whom MID, confirmed by psychological assessment, was the reason for calling upon any of these services.^13^ This is the largest available national data-set on Dutch individuals with MID based on Statistics Netherlands research commissioned by the Ministry of Health.^14^ This linkage allowed the generation of two MID subgroups: one with and one without an intellectual disability registration in the original mental health service database (Fig. 1). The comparison group (no intellectual disability) consisted of all other mental health patients without a (mild) intellectual disability registration in any of the databases used. We included persons aged 18 years or older in 2015 who were available for at least 1 year of follow-up and completed their trajectory before 31 December 2017. Patients aged 75 years and older were excluded because of a low prevalence of MID in the study registries, and thus limited possibility for comparing these age groups.

This study used pseudonymised non-public microdata, which under certain conditions are accessible for statistical and scientific research from Statistics Netherlands.^11^ The authors assert that all procedures contributing to this work comply with the ethical standards of the relevant national and institutional committees on human experimentation and with the Helsinki Declaration of 1975, as revised in 2008. The study protocol was reviewed and approved by the Radboud University Medical Center Institutional Review Board, which waived need for patient consent (approval number 2017-3921). Results are reported in accordance with the Reporting of Studies Conducted using Observational Routinely Collected Data (RECORD) statement.^15^

### Outcomes

The primary outcomes of this study consist of the prevalence of a range of mental health disorders and characteristics of mental healthcare provided. Reported primary diagnoses, based on the DSM-IV criteria, were taken from the submitted insurance claims in the SN-MH service database and converted by Statistics Netherlands into one of 16 predefined diagnostic groups. An overview of the diagnostic groups and associated primary diagnosis are presented in Supplementary Table 1 available at https://doi.org/10.1192/bjo.2023.31. Reported contributing diagnoses were searched to determine the presence of an intellectual disability in the original mental health service files.

A mental health trajectory, defined as an episode of care in which a patient receives mental healthcare for a specific diagnosis within a specific period of time, was initiated for each primary diagnosis. The mental health trajectories include information on the specific setting in which care was provided, the start and end dates of the trajectory and the care activities within the trajectory. We used this information to calculate the duration of mental health trajectories and counted the frequency of various care activities. The duration of trajectories and the number of care activities provide an impression of the intensity and nature of care provided in mental health services. The mental healthcare setting (generic long- or short-term care, elder care, addiction care or forensic care) provides information on the type of care settings in which patients with MID are most likely to receive care. Care activities were segregated into those with direct and those with indirect patient involvement. Direct patient care included diagnostic, treatment or guidance activities. Regarding treatment activities, therapeutic interventions like psychotherapy or pharmacotherapy were listed separately, as they are important treatment models in mental healthcare and often topics of discussion concerning their applicability in patients with MID.^5^ Indirect patient care consisted of coordination of care, time consumed by non-attendance, interprofessional consultations in and outside of the mental health service setting and legal proceedings activities. Indirect patient care was included as an outcome of this study on the assumption that indirect patient care accounts for a larger proportion of the total care provided to patients with MID than to other mental health patients, which was made from clinical experience (authors K.P.M.P., M.M. and J.W.). In addition, interprofessional collaboration, reflected in interprofessional consultations, is considered a way to improve mental health outcomes in patients with intellectual disability.^16,17^ Finally, crisis interventions and hospital admissions were included, as they have been used in previous research to express (mental) health differences between groups.^3^

### Statistical analysis

Variables for all groups were calculated as frequencies, in percentages, or means with s.d. The mean duration of mental health trajectories was calculated as the mean of all trajectories per study group. Comparisons were made between the MID group and the no intellectual disability group, and between the MID subgroup without intellectual disability registration in the mental health service database and the no intellectual disability group. Differences between these groups were tested for statistical significance by *χ*^2^-tests for categorical variables and *t*-tests. The relation between MID and outcomes in mental health services was analysed by logistic regression modelling, controlling for age and gender, and presented as odds ratios with 95% confidence intervals. *P*-values <0.05 were considered statistically significant. Analyses were conducted in SPSS for Windows, version 25.0.

## Results

In the mental health service database, data were available for 337 460 eligible patients, of whom 7596 were identified as having an MID. These patients were more often male (57.5 *v*. 46.2%) and younger (32.5 *v*. 40.5 years), compared with patients without intellectual disability (*n* = 329 864). In total, 60.6% of the patients with an MID (*n* = 4605) had no record of an intellectual disability status in the mental health service database (Table 1).

**Table 1:** Demographics of groups

		No intellectual disability	MID	MID with no intellectual disability registration
Total		329 864	7596	4605
Men, *n* (%)		152 305 (46.2)	4371 (57.5)*^a^	2616 (56.8)*^a^
Age, mean (s.d.)		40.5 (14.4)	32.5 (12.8)*	32.6 (13.0)*
Age groups, *n* (%)	18–24 years	52 246 (15.8)	2968 (39.1)**^b^	1853 (40.2)**^b^
	25–49 years	183 764 (55.7)	3560 (46.9)	2077 (45.1)
	50–74 years	93 854 (28.5)	1068 (14.1)	675 (14.7)

MID, mild intellectual disability.

a. MID/MID with no intellectual disability registartion compared with no intellectual disability.

b. Age group distribution for MID/MID with no intellectual disability registartion compared with no intellectual disability.

*P < 0.001, **P < 0.001 for age group distribution.

Eight of the 16 predefined diagnostic groups were significantly more prevalent in patients with MID (Table 2), with the highest odds ratio for ‘schizophrenia and other psychotic disorders’ (odds ratio 2.06, 95% CI 1.90–2.22) and ‘other childhood disorders’ (odds ratio 2.77, 95% CI 2.02–3.81). Seven diagnostic groups were less prevalent in patients with MID, with low odds ratios for ‘depressive disorders’ (odds ratio 0.46, 95% CI 0.43–0.50) and ‘personality disorders’ (odds ratio 0.41, 95% CI 0.37–0.45). In the MID subgroup without intellectual disability registration, more patients had a ‘no or an unknown diagnosis’ compared with patients without intellectual disability (odds ratio 2.67, 95% CI 2.50–2.84).

**Table 2:** Period prevalence of mental health disorders

	No intellectual disability, *n* = 329 864	MID, *n* = 7596	Odds ratio (95% CI)^a^	MID with no intellectual disability registration, *n* = 4605	Odds ratio (95% CI)^b^
Other childhood disorders, *n* (%)	441 (0.1)	43 (0.6)	2.77 (2.02–3.81)**	17 (0.4)	1.85 (1.13-3.00)*
Schizophrenia and other psychotic disorders, *n* (%)	17 179 (5.2)	827 (10.9)	2.06 (1.90–2.22)**	361 (7.8)	1.56 (1.39-1.74)**
Other diagnosis, *n* (%)	12 615 (3.8)	605 (8.0)	1.82 (1.67–1.98)**	298 (6.5)	1.49 (1.32-1.68)**
No or unknown diagnosis, *n* (%)	85 821 (26.0)	2841 (37.4)	1.78 (1.71–1.90)**	1958 (42.5)	2.67 (2.50-2.84)**
Pervasive development disorders, *n* (%)	10 010 (3.0)	573 (5.7)	1.77 (1.62–1.94)**	257 (5.6)	1.30 (0.15–1.48)**
Neurocognitive disorders, *n* (%)	6599 (2.0)	101 (1.3)	1.67 (1.36–2.06)**	69 (1.5)	1.84 (1.43–2.37)**
Other substance-related disorders, *n* (%)	16 516 (5.0)	750 (9.9)	1.40 (1.30–1.52)**	408 (8.9)	1.31 (1.18–1.46)**
Alcohol-related disorders, *n* (%)	17 861 (5.4)	441 (5.8)	1.13 (1.03–1.25)*	220 (4.8)	0.96 (0.84–1.11)
Anxiety disorders, *n* (%)	60 356 (18.3)	1,242 (16.4)	0.85 (0.79–0.90)**	696 (15.1)	0.77 (0.71–0.83)**
Bipolar and related mood disorders, *n* (%)	11 167 (3.4)	163 (2.1)	0.73 (0.63–0.86)**	62 (1.3)	0.49 (0.38–0.63)**
Attention deficit and conduct disorders, *n* (%)	16 885 (5.1)	320 (4.2)	0.55 (0.49–0.62)**	160 (3.5)	0.44 (0.38–0.52)**
Depressive disorders, *n* (%)	74 672 (22.6)	811 (10.7)	0.46 (0.43–0.50)**	443 (9.6)	0.42 (0.38–0.46)**
Feeding and eating disorders, *n* (%)	5442 (1.6)	70 (0.9)	0.45 (0.36–0.57)**	49 (1.1)	0.52 (0.39–0.69)**
Personality disorders, *n* (%)	45 195 (13.7)	541 (7.1)	0.41 (0.37–0.45)**	327 (7.1)	0.43 (0.38–0.48)**
Somatic symptom disorders, *n* (%)	14 405 (4.4)	115 (1.5)	0.39 (0.32–0.47)**	85 (1.8)	0.47 (0.38–0.58)**
Other problems that are a reason for concern, *n* (%)	383 (0.1)	<10		<10	

MID, mild intellectual disability.

*P < 0.05, **P < 0.001.

a. MID versus no intellectual disability.

b. MID without intellectual disability registration compared with no intellectual disability.

In Table 3, we present an overview of the care provided. Patients with MID had slightly more mental health trajectories (1.5 *v*. 1.4), but were provided with significantly shorter mental health trajectories than patients without intellectual disability (286.8 *v*. 325.7 days). Trajectories were particularly short for patients in the MID subgroup without intellectual disability registration (252.9 days). Compared with patients without intellectual disability, patients with MID were more likely to receive care in an addiction or forensic setting (addiction: odds ratio 1.19, 95% CI 1.11–1.27; forensic: odds ratio 1.81, 95% CI 1.62–2.03) and less likely to receive care in a generic short-term setting (odds ratio 0.76, 95% CI 0.72–0.80). Significantly fewer patients with MID were provided diagnostic (74.9 *v*. 79.7%) and treatment activities (67.7 *v*. 78.0%) compared with patients without intellectual disability; in particular, fewer patients were provided psychotherapy (13.5 *v*. 31.0%). In addition, if psychotherapy was started, the mean number of psychotherapy activities per patient with MID was significantly lower (11.8 *v*. 19.3 activities per patient). The differences compared with the no intellectual disability group were all more prominent in the MID subgroup without intellectual disability registration, with even fewer diagnostic (73.7%), treatment (59.9%) and/or psychotherapy (12.7%) activities.

**Table 3:** Care provided

		No intellectual disability, *n* = 329 864	MID, *n* = 7596	Odds ratio (95% CI)^a^	MID with no intellectual disability registration, *n* = 4605	Odds ratio (95% CI)^b^
Mental health trajectory						
Number of mental health trajectories per patient during research period, mean (s.d.)		1.4 (0.8)	1.5 (1.0)*		1.4 (0.8)	
Cumulative durations of mental health trajectories per patient, in days, mean (s.d.)		325.7 (236.9)	286.8 (236.5)**		252.9 (218.4)**	
Treatment setting, *n* (%)						
Adult care long		95 428 (28.9)	2370 (31.2)	1.03 (0.98–1.09)	1215 (26.4)	0.84 (0.79–0.90)**
Adult care short		210 444 (63.8)	4713 (62.0)	0.76 (0.72–0.80)**	3022 (65.6)	0.95 (0.90–1.02)
Elderly care		19 171 (5.8)	124 (1.6)	1.09 (0.88–1.35)	91 (2.0)	1.30 (1.00–1.68)
Addiction care		30 123 (9.1)	974 (12.8)	1.19 (1.11–1.27)**	545 (11.8)	1.13 (1.03–1.24)*
Forensic		6520 (2.0)	359 (4.7)	1.81 (1.62–2.03)**	169 (6.4)	1.50 (1.28–1.75)**
Direct patient care, *n* (%)						
Diagnostic activities		262 445 (79.7)	5684 (74.9)	0.71 (0.67–0.75)**	3387 (73.7)	0.66 (0.62–0.71)**
Treatment activities		256 910 (78.0)	5135 (67.7)	0.56 (0.53–0.59)**	2751 (59.9)	0.40 (0.37–0.42)**
Psychotherapy activities	*n* (%)	101 918 (31.0)	1024 (13.5)	0.33 (0.30–0.35)**	585 (12.7)	0.30 (0.28–0.33)**
	Mean number per person (s.d.)	19.3 (40.3)	11.8 (22.5)**		12.3 (20.7)**	
Pharmacotherapy activities		112 981 (34.3)	2371 (31.3)	0.88 (0.84–0.92)**	1 238 (26.9)	0.71 (0.67–0.76)**
Supportive care activities		24 213 (7.4)	588 (7.8)	1.10 (1.00–1.19)*	317 (6.9)	0.96 (0.86–1.08)
Indirect patient care, *n* (%)						
Coordination of care activity	*n* (%)	188 983 (64.1)	5,268 (73.4)	1.58 (1.51–1.66)**	2907 (68,0)	1.20 (1.13–1.27)**
	Mean number per person (s.d.)	17.0 (50.1)	21.1 (60.2)**		17.5 (57.2)	
Interprofessional consultation activity, internal	*n* (%)	277 695 (94.1)	6867 (95.7)	1.61 (1.49–1.74)**	4035 (94.4)	1.21 (1.11–1.32)**
	Mean number per person (s.d.)	115.1 (300.9)	134.9 (318.3)**		104.9 (262.9)	
Interprofessional consultation activity, external	*n* (%)	124 194 (42.1)	4142 (59.1)	2.06 (1.97–2.16)**	2276 (53.3)	1.68 (1.58–1.78)**
	Mean number per person (s.d.)	6.2 (12.0)	9.5 (16.3)**		7.5 (13.7)**	
Legal activity		11 272 (3.8)	494 (6.9)	2.00 (1.82–2.19)**	222 (5.2)	1.55 (1.27–1.67)**
Non-attendance activity		27 183 (9.2)	739 (10.3)	1.17 (1.08–1.26)**	406 (9.5)	1.05 (0.95–1.16)
Crisis intervention	*n* (%)	68 489 (20.8)	2501 (32.9)	2.00 (1.90–2.10)**	1479 (32.1)	1.93 (1.81–2.05)**
	Mean number per person (s.d.)	6.7(9.0)	6.4 (10.6)		5.6 (7.7)**	
Mental health hospital admission	*n* (%)	45 001 (13.6)	1551 (20.4)	1.72 (1.63–1.82)**	698 (15.2)	1.20 (1.10–1.30)**
	Mean admission time per admission in days (s.d.)	36.3 (55.0)	39.2 (61.8)		54.7 (76.9)	

Mental health trajectory indicates the period in which patients receives mental healthcare for a specific diagnosis in a mental health service. Direct patient care refers to activities with direct patient involvement, whereas indirect patient care refers to activities without direct patient involvement. Internal interprofessional consultation activity refers to consultations between professionals inside the mental health service setting whereas external activity refers to consultations between professionals outside of the mental health service setting. MID, mild intellectual disability.

*P < 0.05, **P < 0.001.

a. MID versus no intellectual disability.

b. MID without intellectual disability registration compared with no intellectual disability.

Patients with MID more often had activities recorded without direct patient involvement, in particular interprofessional consultations outside the mental health service setting (59.1 *v*. 42.1%) and activities concerning legal affairs (6.9 *v*. 3.8%), compared with patients with no intellectual disability. In addition, when internal or external interprofessional consultation was required within a mental health trajectory, the mean number of consultations per patient with MID was higher (134.9 *v*. 115.1 internal and 9.5 *v*. 6.2 external interprofessional consultations per patient, respectively).

Patients with MID were more likely to require a crisis intervention or mental health hospital admission compared with patients without intellectual disability (crisis intervention: odds ratio 2.00, 95% CI 1.90–2.10; hospital admission: odds ratio 1.72, 95% CI 1.63–1.82), including those patients for whom the intellectual disability was unregistered (crisis intervention: odds ratio 1.93, 95% CI 1.81–2.05; hospital admission: odds ratio 1.20, 95% CI 1.10–1.30).

## Discussion

This is the first population-based database study to focus on patients with MID in advanced mental health services. Patients with MID, compared with patients without intellectual disability, were diagnosed with different mental health disorders and more often received treatment in specialised mental health service settings, such as forensic or addiction care. The mental health trajectories were shorter and the mental health service professionals performed fewer diagnostic and treatment activities in patients with MID, in particular in those cases where the intellectual disability was unregistered, while conducting significantly more indirect patient care activities. At the same time, crisis interventions and mental health hospital admissions were more frequent in patients with MID. These findings are indications of undertreatment in patients with MID, which is likely to result in poor mental health outcomes. A total of 60% of the patients with MID had no intellectual disability registration in their mental health service files. Through linkage with information on MID registration from other sources, we were able to include them in this study.

### Comparison with existing literature

The observed differences in the prevalence of mental health disorders in patients with MID compared with patients with no intellectual disability, including ‘schizophrenia and other psychotic disorders’ (10.9 *v*. 5.2%), ‘depressive disorders’ (10.7 *v*. 22.6%) and ‘personality disorders’ (7.1 *v*. 13.7%), are consistent with other research in mental health services.^7,16–18^ There are several possible explanations for these prevalence differences, including an aetiological origin. However, misinterpretation or mis-categorisation of symptoms by referring health professionals, such as the general practitioner and mental health service professionals, most likely contribute to differences in prevalence; impaired communication and symptom presentation can make it more difficult to categorise using the DSM criteria.^19^ The high prevalence of ‘no or unknown diagnosis’ in patients with MID (37.4%) is an extra indication in this respect.

Adequate recognition of MID also appeared difficult in mental healthcare. The under-registration of MID in our data, which was also found in other research,^7,16^ can be seen as a clear indication of under-recognition of MID in mental health services. The presence of an MID should be taken into account during the whole mental health trajectory, as it is considered a significant risk factor for developing chronic and more severe mental health problems.^16,17^ The under-recognition of MID in combination with the observed indications of undertreatment in patients with MID in our study is therefore worrying. Undertreatment within mental health services can lead to more chronic mental health problems and may partly explain a high prevalence of MID in long-stay wards,^17^ but also contributes to high care use in general, illustrated by high primary care consumption^20^ and emergency department visits^21^ by patients with combined MID and mental health problems.

Indeed, observed signs of undertreatment are more prominent in patients whose intellectual disability was unregistered. This undertreatment is reflected in shorter mental health trajectories (252.9 *v*. 325.7 days) and fewer diagnostic (73.7 *v*. 79.7%) and treatment (59.9 *v*. 78.0%) activities compared with patients with no intellectual disability. A systematic review of studies in addiction care settings also indicated undertreatment in people with MID.^22^ Compared with patients without intellectual disability, they were less likely to initiate and engage in treatment for substance misuse, were more likely to drop out of treatment and treatment was often not adapted to their intellectual capacities. Research in patients with borderline intellectual disability (IQ 70–84) and mental health problems showed that these patients also were less likely to receive treatment compared with adults with no intellectual disability.^23^ In this light, the higher prevalence of mental health hospital admissions (20.4%) and crisis interventions (32.9%) in our study, confirmed in earlier research,^16,23^ are extra disconcerting findings. Although these could also be reflections of more severe and complex mental health problems experienced by patients with MID, following this reasoning we should also have observed longer mental health trajectories and a higher occurrence of treatment activities in patients with MID. However, both of these aspects are contradictory to our findings. High psychotropic use in people with intellectual disability, well-known from previous research,^24,25^ may serve as an additional indication of undertreatment in this group. Unfortunately, the registered pharmacotherapy activities in our study contained no information about patients’ true psychotropic use, and we could not confirm previous results.

Our results show that collaboration, reflected in the high number of interprofessional consultations, is often required in mental health services, especially regarding patients with MID. Despite the fact that collaboration is seen as a tool to improve (mental) healthcare and may reduce hospital admissions and costs,^4,16,26–28^ interprofessional collaboration and other forms of indirect patient care in mental health services have not previously been the subject of a database study.

### Implications for research and/or practice

There is growing awareness within mental health services of the high prevalence of mental health disorders in people with MID and the different care needs of this group. Country-specific guidelines have been developed for these patients,^29,30^ and some examples of good practices are noticed,^4,31–33^ as well as increasing attention on adapted treatment modules in research.^22,34^ Nevertheless, judging from the results of our study, further steps to improve the mental healthcare for people with MID are needed, not only at care professional level, but also at a scientific, organisational and policy level.

Mental health service professionals should be aware of the importance of identifying and registering MID, primarily for good care provision, but also for research purposes. Recurrent education programmes on this topic for mental health service professionals can be an important tool to improve this awareness. Also, screening for intellectual disability in the intake procedure can help to identify an MID at an early stage, enabling adjustments in communication and diagnostic and treatment protocols from the start. Some useful intellectual disability screening tools developed for this purpose are applicable in mental health services, but they need to be further implemented in daily practice.^17,35^ At a scientific level, adequate intellectual disability registration enables researchers to learn in more detail about patient, care professional, and organisation-related factors that contribute to observed differences in health and care provided to patients with MID and without intellectual disability. Given the diagnostic classification difficulties indicated by our study, further research is needed on the applicability of classification instruments for mental health symptoms in patients with MID. In so doing, it is important to focus not solely on the DSM, but also on alternatives, such as the *Diagnostic Criteria for Psychiatric Disorders for Use with Adults with Learning Disabilities* (DC-LD),^36^ *Diagnostic Manual Intellectual Disability* (MD-ID-2)^37^ and Dôsen's integrative approach.^38^ Finally, also at organisational and policy level, steps need to be taken to improve the quality of mental health services for patients with MID. In daily practice, this can already be achieved by using existing instruments like The Green Light Toolkit, an audit instrument for mental health services developed in the UK,^39^ to improve the accessibility of mental health services, but investment in initiatives on optimal, country-specific, collaborative mental health healthcare models, including incentives for payment, will also contribute to this matter.

The focus of our study was advanced mental health services. We expect that differences in mental healthcare between people with and without MID will most likely also be present in basic mental health services. Therefore, this setting deserves attention in future research.

### Strengths and limitations

An important strength of this study is the unique focus on people with MID. By linking the SN-MH service database with the SN-MID database, we were able to identify and include people with MID who were not recorded as such in mental health services. This fills a blind spot regarding the large number of people with MID who would have been overlooked without data linkage.

The use of databases containing routinely collected (health) data, however, also comes with some limitations. First, the mental health service database has limited details of diagnoses and treatments, as the main purpose of the data is to enable proper functioning of the health insurance system. Therefore, only information about the prevalence of main diagnostic groups could be determined, not allowing precise comparisons with international evidence. Second, we excluded patients whose mental health trajectory end date was missing or whose trajectory was not completed within the period observed in this study. Potentially, this concerned patients with more chronic mental health disorders and additional care needs, which might be more prevalent among patients with MID. However, the MID prevalence was lower in the excluded patient group compared with the included patient group (1.9 *v*. 2.3%; Fig. 1). Therefore, we do not think that this has led to a disproportionate exclusion of patients with MID and, consequently, an underestimation of the care provided to those patients in comparison with patients without intellectual disability. Third, the SN-MID database is composed mostly of users of work-related social services, resulting in an under-representation of children and older people. Therefore, we had to restrict our study population to 18- to 75-year-olds. Finally, the SN-MID database contains no exact information on individual-level intellectual and adaptive functioning. However, service use as captured by the SN-MID database is possible only when evidence for an MID diagnosis is provided by psychological assessment when applying for any of these services. As the exact level of intellectual disability is not recorded in the SN-MID database, we cannot entirely rule out that some people with more severe intellectual disability or borderline intelligence were part of our MID group. However, the SN-MID database is currently the best source available in The Netherlands to identify people with MID. This makes this study unique and valuable, as it is the first population-based database study to focus on the care of people with MID in mental health services.

In conclusion, we can say that MID is very often not registered in mental health services and most likely insufficiently taken into account in the trajectories in mental health services. People with MID were diagnosed with different mental health problems and, particularly in those cases in which the intellectual disability was unregistered, provided with fewer diagnostic and treatment activities in mental health services, compared with people without intellectual disability. This is an indication of undertreatment, likely contributing to the high number of crisis interventions and mental health hospital admissions. This underlines the relevance of improving the quality of mental healthcare for people with MID, which can be achieved by creating more professional awareness and (evidenced-based) knowledge of the importance of timely MID recognition and adjustments needed in diagnostics and treatment protocols. In addition, actions are needed at an organisational and policy level to create accessible and effective mental health services for patients with MID.

## Data Availability

Aggregated data from the databases used in this study are publicly available on a dedicated website of Statistics Netherlands (http://statline.cbs.nl). The non-public microdata used to link databases are, under certain conditions, accessible for statistical and scientific research (fees apply). Procedures can be found at www.cbs.nl (for further information, contact microdata@cbs.nl).

## Supplementary material

For supplementary material accompanying this paper visit http://doi.org/10.1192/bjo.2023.31. click here to view supplementary material
