# Supplementary material for: Mental healthcare for adults with mild intellectual disabilities: population-based database study in Dutch mental health services
Source: BJPsych Open. 2023 Mar 3;9(2):e48. doi: 10.1192/bjo.2023.31 (PMC10044331; doi:10.1192/bjo.2023.31)
Supplement: Supplementary file 1 [file S2056472423000315sup001.docx]

| **Supplementary table A: Diagnostic groups** | | |
| --- | --- | --- |
| **DIS code^a^**  **DSM-IV** | **Description DIS code^a^** | **Diagnostic groups pre-defined**  **by Statistics Netherlands** |
| *as1_1* | *disorders with onset usually occurring in childhood and adolescence* |  |
| as1_1.01 | Specific developmental disorders of scholastic skills | Other disorders of childhood |
| as1_1.02 | Specific developmental disorder of motor function | Other disorders of childhood |
| as1_1.03 | Specific developmental disorders of speech and language | Other disorders of childhood |
| as1_1.04 | Pervasive developmental disorders | Pervasive development disorders |
| as1_1.05 | Attention-deficit hyperactivity disorders | Attention deficit and conduct disorders |
| as1_1.06 | feeding disorders of infancy and childhood | Other disorders of childhood |
| as1_1.07 | Tic disorder | Other disorders of childhood |
| as1_1.08 | Enuresis/ Encopresis | Other disorders of childhood |
| as1_1.09 | Other behavioral and emotional disorders with onset usually occurring in childhood and adolescence | Other disorders of childhood |
| *as1_2* | *Delirium, dementia, amnesia and other cognitive disorders* |  |
| as1_2.01 | Delirium | Neurocognitive disorders |
| as1_2.02 | Dementia | Neurocognitive disorders |
| as1_2.03 | Amnestic disorder | Neurocognitive disorders |
| as1_2.04 | Other cognitive disorders | Neurocognitive disorders |
| *as1_3* | *Other mental disorders due to known physiological condition* |  |
| as1_3.01 | Catatonic disorder due to known physiological condition | Other diagnoses |
| as1_3.02 | Personality and behavioral disorders due to known physiological condition | Other diagnoses |
| as1_3.03 | Unspecified mental disorder due to known physiological condition | Other diagnoses |
| *as1_4* | *Mental and behavioral disorders due to psychoactive substance use* |  |
| as1_4.01 | Alcohol related disorders | Alcohol related disorders |
| as1_4.02 | Amphetamine related disorders | Other substance related disorders |
| as1_4.03 | Caffeine related disorders | Other substance related disorders |
| as1_4.04 | Cannabis related disorders | Other substance related disorders |
| as1_4.05 | Cocaine related disorders | Other substance related disorders |
| as1_4.06 | Hallucinogen related disorders | Other substance related disorders |
| as1_4.07 | Inhalant related disorders | Other substance related disorders |
| as1_4.08 | Nicotine related disorders | Other substance related disorders |
| as1_4.09 | Opioid related disorders | Other substance related disorders |
| as1_4.10 | Fencyclidine related disorders | Other substance related disorders |
| as1_4.11 | Sedative, hypnotic, or anxiolytic related disorders | Other substance related disorders |
| as1_4.12 | Multiple psychoactive substance related disorders | Other substance related disorders |
| as1_4.13 | Other psychoactive substance related disorders | Other substance related disorders |
| *as1_5* | *Schizophrenia and other psychotic disorders* |  |
| as1_5.01 | Schizophrenia | Schizophrenia and other psychotic disorders |
| as1_5.02 | Schizophreniform disorder | Schizophrenia and other psychotic disorders |
| as1_5.03 | Schizoaffective disorders | Schizophrenia and other psychotic disorders |
| as1_5.04 | Delusional disorders | Schizophrenia and other psychotic disorders |
| as1_5.05 | Brief psychotic disorder | Schizophrenia and other psychotic disorders |
| as1_5.06 | Shared psychotic disorde | Schizophrenia and other psychotic disorders |
| as1_5.07 | Psychotic disorder due to known physiological condition | Schizophrenia and other psychotic disorders |
| *as1_6* | *Mood disorders* |  |
| as1_6.01 | Depressive disorders | Depressive disorders |
| as1_6.02 | Bipolar and related mood disorders | Bipolar and related mood disorders |
| *as1_7* | *Anxiety and stress related disorders* |  |
| as1_7.01 | Panic disorder without agoraphobia | Anxiety disorders |
| as1_7.02 | Panic disorder with agoraphobia | Anxiety disorders |
| as1_7.03 | Agoraphobia without panic disorder | Anxiety disorders |
| as1_7.04 | Specific (isolated) phobias | Anxiety disorders |
| as1_7.05 | Social phobias | Anxiety disorders |
| as1_7.06 | Obsessive-compulsive disorder | Anxiety disorders |
| as1_7.07 | Post-traumatic stress disorder | Anxiety disorders |
| as1_7.08 | Acute stress reaction | Anxiety disorders |
| as1_7.09 | Generalized anxiety disorder | Anxiety disorders |
| as1_7.10 | Anxiety disorder due to known physiological condition | Anxiety disorders |
| as1_7.11 | Substance related anxiety disorder | Anxiety disorders |
| as1_7.12 | Anxiety disorder, unspecified | Anxiety disorders |
| *as1_8* | *Somatoform disorders* |  |
| as1_8.01 | Somatization disorder | Somatic symptom disorders |
| as1_8.02 | Undifferentiated somatoform disorder | Somatic symptom disorders |
| as1_8.03 | Conversion disorders | Somatic symptom disorders |
| as1_8.04 | Pain disorders related to psychological factors | Somatic symptom disorders |
| *as1_10* | *Dissociative and conversion disorders* |  |
| as1_10.01 | Dissociative amnesia | Other diagnosis |
| as1_10.02 | Dissociative fugue | Other diagnosis |
| as1_10.03 | Dissociative identity disorder | Other diagnosis |
| as1_10.04 | Depersonalization-derealization syndrome | Other diagnosis |
| as1_10.05 | Dissociative and conversion disorder, unspecified | Other diagnosis |
| *as1_11* | *Sexual dysfunction* |  |
| as1_11.01 | Hypoactive sexual desire disorder | Other diagnosis |
| as1_11.02 | Sexual arousal disorders | Other diagnosis |
| as1_11.03 | Orgasmic disorder | Other diagnosis |
| as1_11.04 | Vaginismus/ Dyspareunia | Other diagnosis |
| as1_11.05 | Sexual dysfunction  due to physiological condition | Other diagnosis |
| as1_11.06 | Sexual dysfunction  due to a substance | Other diagnoses |
| as1_11.07 | Paraphilias | Other diagnoses |
| as1_11.08 | Gender identity disorders | Other diagnoses |
| as1_11.09 | Unspecified sexual dysfunction | Other diagnoses |
| *as1_12* | *Eating disorders* |  |
| as1_12.01 | Anorexia nervosa | Feeding and eating disorders |
| as1_12.02 | Bulimia nervosa | Feeding and eating disorders |
| as1_12.03 | Eating disorder, unspecified | Feeding and eating disorders |
| *as1_13* | *Sleep disorders* |  |
| as1_13.01 | Primary sleep disorders | Other diagnoses |
| as1_13.02 | sleep disorders due to other mental disorder | Other diagnoses |
| as1_13.03 | Other sleep disorders not due to a substance or known physiological condition | Other diagnoses |
| as1_13.04 | Other sleep disorders not due to a substance | Other diagnoses |
| as1_13.07 | Sleep disorder, unspecified | Other diagnoses |
| *as1_14* | *Impulse disorders* |  |
| as1_14.01 | Intermittent explosive disorder | Other diagnoses |
| as1_14.02 | Kleptomania | Other diagnoses |
| as1_14.03 | Pyromania | Other diagnoses |
| as1_14.04 | Pathological gambling | Other diagnoses |
| as1_14.05 | Trichotillomania | Other diagnoses |
| as1_14.06 | Impulse disorder, unspecified | Other diagnoses |
| as1_14.07 | Other impulse disorders | Other diagnoses |
| *as1_15* | *Adjustment disorders* |  |
| as1_15.01 | Adjustment disorders | Conducting disorders^b^ |
| *as1_17* | *Other problems that are a reason for concern* |  |
| as1_17.01 | Psychological and behavioral factors associated with disorders or diseases classified elsewhere | Other problems that are a reason for concern |
| as1_17.02 | Drug-induced extrapyramidal and movement disorders | Other problems that are a reason for concern |
| as1_17.03 | Unspecified adverse effect of drug or medicament | Other problems that are a reason for concern |
| as1_17.04 | problems related to primary support group | Other problems that are a reason for concern |
| as1_17.05 | Problems related to abuse or neglect | Other problems that are a reason for concern |
| as1_17.06 | Additional problems that are a reason for concern | Other problems that are a reason for concern |
| *as1_18* | *Additional code/no diagnosis* |  |
| as1_18.01 | Unspecified mental disorder | Other diagnoses |
| as1_18.02 | No diagnose | Other diagnoses |
| as1_18.03 | Diagnose postponed | Other diagnoses |
| *as1_19* | *Primary childhood disorder* |  |
| as1_19.01 | Post-traumatic stress disorder, childhood | Other diagnoses |
| as1_19.02 | Affective disorders, childhood | Other diagnoses |
| as1_19.03 | Adjustment disorders, childhood | Other diagnoses |
| as1_19.04 | Regulation disorders, childhood | Other diagnoses |
| as1_19.05 | Sleep disorders, childhood | Other diagnoses |
| as1_19.06 | Eat disorders, childhood | Other diagnoses |
| as1_19.07 | Relation and communication disorder, childhood | Other diagnoses |
| *as2_16* | *Personality disorders* |  |
| as2_16.01 | Cluster A personality disorders | Personality disorders |
| as2_16.02 | Cluster B personality disorders | Personality disorders |
| as2_16.03 | Cluster C personality disorders | Personality disorders |
| as2_16.04 | Personality disorders, unspecified | Personality disorders |

^a^ DIC code = represents DIagnosis treatment Combination products that can be claimed by the Dutch insurance compagnies. The codes are determined by the Dutch Healthcare authority and are linked to diagnoses established in *Diagnostic and Statistical Manual of Mental Disorders, 4^th^ edition* (DSM-IV); ^b^ Due to changes in financing this diagnostic group conducting disorder’ is no longer used and therefore not taken into account in this study
